# Formulation of Ready-to-Eat Soup for the Elderly: Nutritional Composition and Storage Stability Study

**DOI:** 10.3390/foods12081680

**Published:** 2023-04-18

**Authors:** Pornrat Sinchaipanit, Anantita Sangsuriyawong, Piyanuch Visetchart, Nilesh Prakash Nirmal

**Affiliations:** Institute of Nutrition, Mahidol University, 999 Phutthamonthon 4 Road, Salaya, Nakhon Pathom 73170, Thailand

**Keywords:** elderly, food product, ready-to-eat soup, storage stability

## Abstract

Lack of appetite is a common problem in elderly people which could lead to the risk of malnutrition. Soup-based product formulation and supplementation for the elderly is an interesting and convenient way to maintain nutritional status. Hence, this study aims to develop ready-to-eat (RTE) soup and instant soup powder using common agricultural commodities. The results indicated that among all formulations, the F7 formula comprised brown rice (15 g), pumpkin (32.5 g), sweetcorn (12.5 g), red tilapia (17.5 g), rice bran oil (1.0 g), and water (21.5 g) with energy ratio (C:P:F) of 58:23:20 receiving the highest sensory scores. The selected formulation (F7) was also transformed into instant powder and both RTE soup and instant powder were evaluated for nutritional composition and storage stabilities at 5 °C and 25 °C, respectively. The nutritional composition analyses indicate that 100 g of RTE soup consists of 13.8 g carbohydrates, 4.9 g proteins, 1.8 g fats, and 1.5 g dietary fibers; the soup is also a rich source of antioxidants and β-carotene. Storage studies suggested that the content of β-carotene and antioxidant activity of both (ready-to-eat and instant powder) types of soup decreased with increasing storage time, while a slight increase in yeast and mold count (<50 cfu/g) was noted. Most importantly, no pathogenic bacteria were detected in ready-to-eat and instant soup during the storage study of 6 weeks at 5 °C and 6 months at 25 °C, respectively. In terms of the high nutritional composition and functional value of the product, 4 weeks of storage at 5 °C and 4 months of storage at room temperature were suggested for ready-to-eat and instant powder soup product, respectively.

## 1. Introduction

The world’s aging population keeps increasing, and WHO predicts that one in six people will be aged over 60 years by 2050 and will reach 2.1 billion [1]. Among these, 80% of the aging population will be from low- and middle-income countries [2]. Nevertheless, meeting the diet and nutrition requirements of older people is the key element to promoting healthy aging and quality of life [3]. The availability of high-dense energy food products in the market is rather expensive since the ultra-process food ingredients and functional elements have been integrated as main materials, for example, soy protein, pea protein, maltodextrin, modified starch, and food additives such as emulsifiers, stabilizers, and antioxidants. Using agricultural commodity replacement to the ultra-processed ingredients in elderly food products not only introduces value-added agricultural products, but also generates the product with acquainted taste and flavor which people consume in daily life. Likewise, a variety of complex carbohydrates and beneficial phytochemicals and the quality of protein and fat are the alternate choice. In addition, this affects economic growth, particularly for food and nutrition security, food availability, accessibility, utilization, and stability corresponding with the Sustainable Development Goals (SDGs).

Healthy eating is simply eating an adequate amount of macro and micronutrients and well-balanced meals to support the body’s needs. A proper meal should contain 45–60% of carbohydrates, 10–15% of proteins, 20–25% of fats, and about 30 g/day of dietary fibers [4]. Protein provides 4 kcal of energy. The role of protein in the body is to provide structure, regulate body processes, and transport materials; in addition, it also curbs hunger and improves satiety. The function of carbohydrates is energy production (4 kcal/g), energy storage, building macromolecules, sparing protein, and assisting in lipid metabolism. Fat serves as an important depot for energy storage, protects internal organs, supports cell growth, keeps cholesterol and blood pressure under control, and helps the body absorb vital nutrients, in particular fat-soluble vitamins. A balanced meal is a snapshot of a diet that covers the three core food groups. An imbalanced diet consists of either an excess or inadequate intake of macronutrients. For example, excessive fat and protein with insufficient carbohydrates including too many calories or too few calories result in metabolite effects and risk for non-communicable diseases or aggravation of an existing disease. Besides this, aging causes many health-related issues such as weakness, and physical, sensorial, and cognitive impairments which result in loss of appetite and consequently quality of life of the person [5]. Additionally, age-related difficulties in swallowing food are another factor affecting the health of elderly people [6,7]. Nevertheless, a healthy diet is imperative for the well-being and health of the elderly. Hence, the preparation of food with textural modification without losing nutritional composition is very important for elderly consumers [8,9].

Soup-based product is a more interesting food preparation solution for the elderly since it can fortify any functional ingredients or micronutrient deficiency to meet the body’s requirements. This will be more beneficial for vulnerable elderly populations and worth preventing and promoting health than curative care. The conventional method and simple technology such as pasteurization and drying process are combined to maintain the quality and stability of food during storage and be accessed for small and medium enterprises including household cooking. Consequently, utilizing the agricultural commodities and common thermal process to develop elderly soup products based on recommended macronutrient requirements was the objective of this research. In addition, the changes in quality, microbiological safety, and antioxidant activity were followed during storage.

## 2. Materials and Methods

### 2.1. Raw Material Collection and Preparation

Brown jasmine rice *(Oryza sativa* L.) (Royal Umbrella™ brand, CP Intertrade Co., Ltd., Bangkok, Thailand), yellow sweet corn kernel *(Zea mays* saccarata) (Doikham™ brand Food Products Ltd., Chiang Rai, Thailand), yellow flesh pumpkin (*Cucurbita moschata*) (Doikham™ brand Food Products Ltd.), high oryzanol rice bran oil (King™ brand, Thai Edible Oil Ltd., Bangkok, Thailand) and fresh red tilapia fish *(Oreochromis* sp.) (CP™ brand, CP Intertrade Co., Ltd.) were purchased from grocery supermarkets in Bangkok, Thailand. Inulin was obtained from Food Ingredient Technology Co., Ltd., Bangkok, Thailand.

The raw materials were separately cleaned with running tap water. Skin, seeds, and inedible parts of the pumpkin were removed. The pumpkin flesh was cut into pieces of about 1 inch in thickness. Corn kernels were removed from the sweet corn cobs. Fish flesh in fillet form was prepared. The prepared materials were steamed over boiled water for approximately 15 min for pumpkin pieces and about 10 min for corn kernels and fish fillets. Only brown jasmine rice was cooked using an electric rice cooker. After that, the cooked materials were cooled to room temperature at 25 °C, and then kept at 5 ± 2 °C for further use.

### 2.2. Formulations and Ready-to-Eat Soup Development

Before the formulation of ready-to-eat soup, all ingredients were analyzed for the content of carbohydrates, protein, fat, dietary fiber, ash, and moisture (Supplementary material). The INMUCAL-Nutrients program V.4.0 [10] developed by the Institute of Nutrition, Mahidol University, Thailand was applied to generate the elderly soup based on the recommended macronutrient energy distribution. The energy distribution ratio of 50–65% carbohydrate, 10–25% protein, and 20–30% fat was key to formulating the elderly soup products [11]. Different soup formulations were developed by varying the incorporation of carbohydrate ingredients; either one, two, or three carbohydrate combinations were used. This generated a total of 7 formulations as presented in Table 1.

The cooked pumpkin, corn kernel, fish, and brown jasmine rice including rice bran oil and water were weighed according to the soup formula (F1–F7). All ingredients were ground together by a homogenizer (Buono Professional, Thailand) at a high sheer speed for 20 min. The mixed soup was pasteurized at 100 °C for 20 min and hot filled in a wide-mouth glass bottle with sealing closure (200 mL), then immediately cooled and kept at refrigerator temperature (5 ± 2 °C) for analysis. All F1–F7 formulas evaluated the degree of enjoyment of product by sensory analysis (5—smiling face, *n* = 30). The soup sample that received the highest score for overall acceptability was selected. The proximate compositions, β-carotene content, antioxidant activity, physicochemical quality, and microbial safety were determined. The stability of ready-to-eat soup products was followed during storage at 5 ± 2 °C for 6 weeks.

#### Dehydrated Soup Powder Preparation

The selected soup formula (F7) was further developed into the instant soup powder. As in the previous study in the laboratory, an optimum of 15% inulin was added to improve the mouthfeel of reconstituted dry powder soup. The blended soup was prepared and then applied onto a pilot-scale double-roller drum dryer (B.W.S. Trading Limited Partnership, Bangkok) 30 cm in diameter, 45 cm in length, with a 0.1 mm roller gap, a 1 rpm roller speed and an 8–9 kg/cm^2^ steam pressure. The drum-dried sheets were collected and ground by passing through a 100-mesh screen sieve. The dehydrated soup powder was packed in close-sealed laminated aluminum foil bags and stored at room temperature (25 ± 2 °C) for further study. The proximate compositions, dietary fiber, β-carotene content, antioxidant activity, physicochemical quality, and microbial safety were determined. The stability of instant soup powder was examined during storage at 25 ± 2 °C for 6 months.

### 2.3. Physico-Chemical Analyses

#### 2.3.1. Proximate Analysis

The protein was analyzed using the Kjeldahl method as mentioned by AOAC 992.23 [12]. Total fat content was determined after acid hydrolysis and solvent extraction using the Soxhlet™ apparatus as mentioned by AOAC 992.06 [12]. Dietary fiber was analyzed by using the enzymatic gravimetric method as mentioned by AOAC 985.29. Ash content was determined by heating a sample in a muffle at 550 °C as mentioned by AOAC 945.46 [12]. Moisture content was measured by drying a sample in a hot-air oven at 105 °C as mentioned by AOAC 927.05 [12]. The ready-to-eat soup or instant soup powder was sampled in triplicate and homogenized before being taken for analysis. Available carbohydrate (including dietary fiber) was assessed by subtracting the sum of the percentage of moisture, total fat, protein, and ash from 100. The energy was calculated by using multiplication factors; 4 for carbohydrate and protein and 9 for fat.

#### 2.3.2. Physicochemical Attributes

Water activity (a_w_) was measured by a portable water activity meter (Novasina Model MS1, Switzerland) at room temperature (25 °C). The pH value was measured using a pH meter (Eco Met P25 Istex, Inc., Seoul, Korea) at room temperature (25 °C). Relative viscosity was determined by a rotational viscometer (Model DV-II, Brookfield, Middleborough, KY, USA) with an RV spindle no.2 at a speed of 1 g under room temperature (25 °C). The color was determined using a colorimeter (Color Flex EZ spectrophotometer, HunterLab, VA, USA) with a pulsed xenon lamp as a light source. The color values were expressed in the CIELAB parameter, where L*, a*, and b* are the lightness, redness, and yellowness, respectively.

#### 2.3.3. β-Carotene Content Determination

The β-carotene content in the sample was determined following Oulai et al. [13] with a slight modification. Three samples of ready-to-eat soup or soup powder were pooled together and homogenized. The homogenized sample was treated with 10% ascorbic acid and 2M potassium hydroxide in methanol. Thereafter, the solution was extracted with hexane (2 times), and the extract was washed with 10% sodium chloride until alkali free. The obtained solution was evaporated using a rotary evaporator at 35 °C. The precipitate was further dissolved in the mobile phase (acetonitrile, methanol, and acetone at a ratio of 60:30:10). The dissolved solution was filtered using a 0.4 mm syringe filter to remove any insoluble debris. The clear solution was injected on HPLC fitted with a C18 column, equipped with a diode array detector at a wavelength of 450 nm. A pure β-carotene crystalline (Sigma-Aldrich, Saint Louis, MO, USA) was also run at the different concentrations of 0.3, 0.6, 1.5, and 2.5 µg/mL for a standard curve. The β-carotene content in the sample was estimated using a standard curve.

#### 2.3.4. Antioxidant Activity Determination

The antioxidant activity of the sample was measured as an oxygen radical absorbance capacity (ORAC). Although there are various antioxidative mechanisms and assays reported in the literature, ORAC has been widely accepted as a standard assay method to determine antioxidant capacity in food, nutraceutical, and pharma industries [14]. Three samples of ready-to-eat soup or soup powder were pooled together and homogenized. Briefly, 500 μL of the sample was added into the fluorescein solution (3.0 mL, 8.2 × 102 μM) and 2,2-azobis [2-amidinopropane] dihydrochloride (500 μL, 153 mM) (Wako Pure Chemical, Japan) and mixed [14]. The reaction mixture was diluted using 75 mM potassium phosphate buffer solution at pH 7.2. The properly diluted solution was read at an excitation wavelength of 493 nm and an emission wavelength of 515 nm using a spectrofluorometer (Perkin Elmer LS 55, USA). The ORAC value of the sample was determined from a standard curve of Trolox (6.25–100 ppm) (Sigma-Aldrich, Saint Louis, MO, USA) and expressed as µg Trolox equivalents (TE) per 100 g.

### 2.4. Microbiological Quality

To prepare the sample dilution, 25 g of the sample was added into 225 mL of 0.1% buffered peptone water (Oxoid Ltd., Hampshire, UK) and blended for 1 min in a stomacher (500 rpm; IUL Instruments, Barcelona, Spain). A serial of decimal dilution was prepared from the above stock solution. Total aerobic bacteria count was enumerated on plate count agar (PCA, Oxoid Ltd.) after incubation at 30 °C for 48 h [15]. Yeast and mold were enumerated on PCA, adding 100 mg chloramphenicol/liter and incubating at 25 °C for 5 d [16]. *Escherichia coli* counts were determined by a presumptive test and then confirmed by using EC broth by incubating at 35 °C for 48 h [17].

For pathogenic microorganisms, *Salmonella* analysis was conducted using the method of ISO 6579-1 (2017). *Staphylococcus aureus*, *Bacillus cereus*, *Clostridium perfringens*, and *Clostridium botulinum* were analyzed following the Bacteriological Analytical Manual (BAM) [18]. The brief experimental procedure for pathogenic microorganism determination has been provided in the Supplementary Materials.

### 2.5. Sensory Evaluation

A balanced incomplete block was designed for sensory analysis. The ready-to-eat soup samples (100 mL) were coded with a 3-digit random number and served warm (about 50 °C) to each subject in random sequences. The semi-trained panelists aged 60 to 70 years (*n* = 30) were appealed to taste the soup and assessed their likeness. A five-point hedonic scale (1—dislike very much, 2—dislike moderately, 3—neither like nor dislike, 4—like moderately, and 5—like very much) was employed for overall acceptability, taste, odor, texture, and color. The protocol of this study was approved by Mahidol University Central Institutional Review Board (Protocol No. MU-CIRB; 2016/187.1011).

### 2.6. Statistical Analysis

The study was performed in triplicate. The analysis was conducted for three replicates and presented the results as mean ± SD. One-way analysis of variance (ANOVA) was used to analyze the data on the SPSS© statistical software (version 19) [19]. Duncan’s Multiple Range test (DMRT) was a post hoc test to measure specific differences between pairs of means with a significant difference level of 0.05.

## 3. Results and Discussion

### 3.1. Formulation and Development of Ready-to-Eat Soup

The nutritional components such as the presence of carbohydrates, dietary fiber, proteins, and fats for any food product are important in terms of health benefits to the consumer. Besides this, accessibility and affordability of the raw materials to prepare healthy food are a vital component. Hence, in this study, we have used common agricultural commodities which are easily available in the market to prepare ready-to-eat soup for the elderly. The proximate analyses of all raw materials indicated that the highest carbohydrate content was presented in sweet corn kernels (35 g/100 g) followed by brown rice (30 g/100 g) and pumpkin (11 g/100 g) (Supplementary Materials). The highest protein content (20 g/100 g) was noted in red tilapia fish fillets and the lowest in pumpkin (1.3 g/100 g). In terms of fat, accepted rice bran oil possesses 100%, while other ingredients range from 0.3 to 2.5 g/100 g. In addition, dietary fiber was higher in sweet corn (5.5 g/100 g) and lowest (1.9 g/100 g) in brown jasmine rice. The proximate analyses of all raw ingredients granted the suitability of these ingredients for the preparation of soup for the elderly. Macronutrients such as carbohydrates, proteins, and fats are known to deliver energy and support normal physical activities in daily life [20]. Besides this, non-nutritive bioactive compounds, in particular the antioxidants, promote health and help to prevent non-communicable diseases (NCDs) and cancer [21,22]. All these nutritive and non-nutritive ingredients govern the final sensory properties such as odor, flavor, taste, and color of the food products [23].

Based on the recommended energy distributions for elderly food, Table 1 represents the different formulation schemes for the preparation of soup. F1–F3 contained a single carbohydrate, F4–F6 comprised two carbohydrates and F7 consisted of all three carbohydrates. The protein source and fat ingredients were similar but in different amounts depending on the formula. The developed soup, F1–F7, carried energy of around 160–215 kcal/200 mL (one serving). Displayed energy distributions of macronutrients in soup formulas, F1–F7, was 57–59% carbohydrate, 21–23% protein, and 20–21% fat. Riyad and Rizak [24] reported that soup for the elderly developed with oat, spinach, broccoli, and bananas covered the recommended dietary allowances for crude fibers, carbohydrates, proteins, fats, and caloric values in the range of 29.3–44.3%, 29.6–30.6%, 14.3–18.5%, 10–12.5% and 9.4–10.7 kcal/cup of soup, while another study was conducted to prepare soup for the elderly containing a high amount of β-carotene (non-nutritive bioactive compound) using a different ratio of pumpkin and carrot [25]. The study indicated that the selected highly preferred soup (pumpkin: carrot, 2:1) consists of 60.88% of carbohydrates, 9.82% of proteins, and 23.21% of fats [25]. The content of carbohydrates, proteins, and fats in the developed soup for the elderly in the present study is concurrent with the previous report of Riyad and Rizak [24] and Irwan [25]. The sensory analyses of all formulated soups were conducted using a five-point hedonic scale (Table 2). Healthy seniors with an average age of 60–70 years old (*n* = 30) voluntarily participated in the soup product liking test. The outcome indicated that F1 had a pale white color and a moderately thick, rice odor. F2 presented the vivid yellow, moderately thick, intense pumpkin flavor and slightly sweet taste. F3 exhibited a yellow color, a mildly thick, sweet smell, and tasted like sweet corn. F4–F6 indicated the yellow color, smooth texture, and slightly sweet taste and odor, similar to the characteristics of their combined carbohydrate ingredients, while F7 revealed a bright yellow color, moderately thick, smooth texture, and sweet odor similar to a combination of distinctive attributes of brown jasmine rice, pumpkin, and sweet corn altogether. Among all formulations, F7 formula received the highest scores for overall acceptability, taste, odor, and texture, which scored over 4.5, meaning a score from “like moderately” to “like very much”, excluding color. F4 was the second most liked formula and had a score of most attributes slightly over 4 (“like moderately”), excluding color score, which was the highest. F5 and F6 revealed the liking score of quality attributes close to 4 or a little bit higher than 4, whereas F1–F3 had the lowest score for all quality attributes compared to the other formulas. Using a combination of carbohydrates in soup products may impact liking score more than using a single carbohydrate source. It is likely that an appropriate blending of both types and the number of carbohydrates may boost the distinctive characteristics, enhance the flavor perception, and mask the sharp odor and strong flavor [26,27]. Based on the highest overall acceptability score, F7 was selected to conduct quality analysis and further study.

Table 3 shows the proximate composition, physicochemical properties, and microbiological quality of the selected ready-to-eat soup formulation. An amount of 100 g of soup consisted of 13.8 g carbohydrates, 4.9 g proteins, 1.8 g fats, 1.5 g dietary fibers, 85.6% moisture content, and carried energy of about 91 kcal. The energy distribution of soup came from carbohydrates in an amount of 60.6%, 17.8% of it came from protein, and 21.5% of energy was generated from fat. Adequate energy intake and energy balance can prevent the development of deficiency diseases and reduce the likelihood of toxicity associated with excess consumption of certain food groups [28]. The macronutrient distributions including sensorial appeal and food palatability were expected for elderly food product development [29,30]. Nevertheless, the developed ready-to-eat soup can be used as a supplement meal or enrich diet for senior people, particularly older people with chewing problems and loss of appetite or reduced eating capability. One serving (200 mL) of developed ready-to-eat soup carries 3 g dietary fiber or over 10% of the daily intake value (30 g) by achieving FDA definitions of “good source of fiber”. An appropriate intake of dietary fiber enabled the gain of benefits from its functions such as reducing the risk of cardiovascular diseases, diverticulosis, constipation, irritable colon, cancer, and diabetes [31,32]. The smooth texture, viscous fluid, vivid yellow color, and slightly acidic pH were obtained for the selected ready-to-eat soup. Additionally, it had rich β-carotene (1560 µg/100 g) and antioxidant activity (ORAC = 3074 µgTE/100 g) due to the natural phytonutrients from raw materials. The functions of ß-carotene are development of vitamin A precursors, antioxidant effects, immune boost, reduction in mutagenesis and tumors including slowing down the age-related macular degeneration and development of cataracts. The recommended intake for β-carotene is about 2.5–2.9 mg/6 mg carotenoids per day [33]. In Germany, 2–4 mg/d of β-carotene is recommended [34]. Consumed the ready-to-eat-soup or instant soup powder may provide β-carotene of approximately 3 mg/1 serving. The health benefit of antioxidants is defense against oxidative and free-radical-mediated reactions, suppression of DNA damage, immune system stimulation, antitumor potential, and prevention the chronic degenerative diseases [22]. These antioxidant functions depend on specific elements and ingredient sources. The ORAC is the one index to verify the general health benefit of products to extend a further experiment. Moreover, the microbial analyses revealed that the soup product had less than 10 cfu/mL of yeast and mold, and less than 1.1 MPN/100 mL of coliform, while no pathogenic bacterial count was detected, which complied with Nation Food Notification [35]. Hence, the developed ready-to-eat soup for the elderly was safe for consumption, more convenient, and helped to reduce the difficulty in the preparation of soup.

### 3.2. Stability of Ready-to-Eat Soup during Refrigerated Storage

Changes in the quality of the product during storage are well correlated to the product expiration or best-before date. Following analysis of physicochemical properties and microbial safety during storage was crucial for an index of product stability. Table 4 shows the physicochemical and microbiological quality of ready-to-eat soup during storage at 5 °C for 6 weeks. As the number of storage days increased, a slight reduction in β-carotene and ORAC value was noted, but they were not significantly different until week 4. Thereafter, significant difference in β-carotene and ORAC value in the ready-to-eat soup was reported at 6 weeks compared to the initial day of storage (*p* < 0.05). The β-carotene content (1561 µg/100 g) from day 0 decreased to 1293 µg/100 g at 6 weeks of storage. The ORAC value decreased from the start of the day (3074 µg TE/100 g) to 2324 µg TE/100 g after 6 weeks of storage. There was no significant (*p* > 0.05) change noted in the pH value (6.51–6.52) of soup during 6 weeks of storage at 5 °C. Soup viscosity seemed slightly reduced, which was about 1.8 × 10^4^ cP at the beginning and revealed 1.6 × 10^4^ cP at the end of storage (*p* ≤ 0.05). Reduced-viscosity soup may be influenced by the rearrangement of amylose molecules since the starch retrogradation occurs during prolonged storage time [36]. The lightness value slightly decreased, but the redness and yellowness values slightly shifted to redness and blueness, respectively. The change in soup color might be the result of the browning reaction caused by the interaction between reducing sugar and free amino acid contained in the used ingredients.

The microbiological indices were followed to assess product safety. Yeast and mold started at < 10 cfu/mL at week 0 and continually increased to <50 cfu/mL at the end of storage. Coliform was < 1.1 MPN/100 mL through storage for 5 weeks and increased to <2.2 MPN/100 mL at the last week of storage. Meanwhile, *E. Coli* (MPN/g) and pathogenic bacteria such as *Salmonella, S*. *cereus*, *B*. *cereus*, and *C. perfringen* were not detected in ready-to-eat soup products during prolonged storage at 5 °C for 6 weeks. The microbial analyses indicate that the ready-to-eat soup product was safe for consumption over the storage period in compliance with the Food Notification [35,37]. Although cold storage could delay the increasing number of survival spoilage microorganisms and limited the chemical reaction that promotes food deterioration, the presence of bioactive compounds should be considered owing to their health benefits. Hence, to compromise the health benefit, quality characteristics, and product safety, it was suggested to keep the ready-to-eat elderly soup product for up to 4 weeks at refrigerator temperature (5 ± 2 °C). Additionally, re-heating of ready-to-eat soup was recommended to obtain a fresh and good taste.

### 3.3. Instant Dehydrated Soup Powder

The common drum dryer traditional technique was used to produce dehydrated soup powder. Inulin (15%) was added to improve the texture and food palatability. Additionally, it provided dietary fiber and increased yield percentage from about 21.5% (0% inulin) to 30.2% (adding 15% inulin). Inulin used in this study was extracted from Jerusalem artichoke tubers. Generally, the amount of inulin present in any food is safe for consumption. However, added or supplemented inulin portion should be regulated to minimize the side effects. It was reported that the intake of 8–18 g of inulin per day is safe for up to 24 weeks [38]. The drum dryer technique is easy to operate, has good rehydration feature, is clean and hygienic, and has high energy efficiency [39]. However, it might affect the body texture after reconstituting the dehydrated food powder, particularly for carbohydrate-based soup products, because the disaggregate of amylose and amylopectin chains become re-associated to form more ordered structures or call starch retrogradation [36].

**Figure 1:**
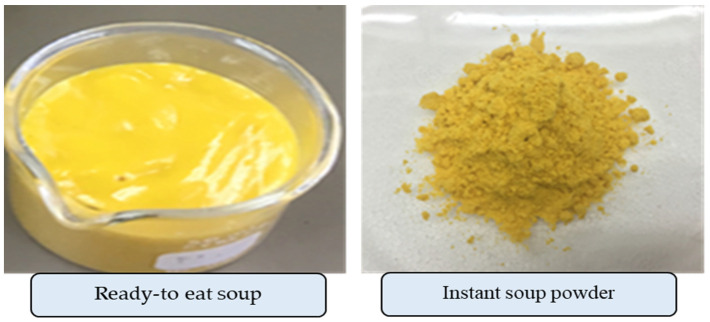
Photographs of Ready-to-eat soup and instant soup powder.

The proximate analyses revealed that 100 g of dehydrated instant soup powder contained 41.4 g carbohydrate, 14.7 g protein, 5.4 g fat, 30.8 g total dietary fiber (insoluble fiber 5.1 g and soluble fiber 25.7 g), 5.1% moisture, and energy of 273 kcal (Table 5). The energy distribution of dehydrated soup powder ranged from 60.6% of carbohydrates, 21.5% of proteins, and 17.8% of fats. The increased amount of fiber reflects the inulin added into the soup. Dietary fiber had a direct impact on gut mobility and prevention of constipation and increase in insulin sensitivity, with reduction in the risk of diabetes mellitus, obesity, chronic inflammation, and colonic cancer [40,41]. Moreover, dietary fiber, in particular soluble fiber, can influence satiety by retarding the digestive enzyme and stimulating the secretion of gut hormone, peptide YY (PYY) and glucagon-like peptide-1 (GLP-1) including leptin and insulin [42]. The dried tiny particles, yellow color, slightly acidic, natural flavor, and odor were observed for the physical appearances of the soup powder product (Table 5). Figure 1 shows a photograph of ready-to-eat soup and instant soup powder. Additionally, the dehydrated soup powder was rich in β-carotene (4918 µg/100 g) and ORAC value (8159 µgTE/100 g). Besides this, the microbiological qualities indicated the presence of yeast and mold in an amount of less than 25 cfu/g and that of *E. coli* of less than 1.1 MPN/g. Meanwhile, no pathogenic bacteria including *Salmonella*, *S. aureus*, *B. cereus*, and *C. perfringens* were detected that complied with the food standard of semi-processed food [43] and pathogenic microorganisms in foods [35], respectively. This confirmed that the instant soup powder prepared using the drum dryer technique under mentioned setting condition retained the nutritive value and safety for consumption.

Reconstitution of dehydrated soup powder was conducted by dissolving 55 g of instant soup powder in 150 mL of boiled water and stirring until dissolved. The reconstituted soup of 100 g contained about 15 g of carbohydrates, 5.6 g of proteins, 1.9 g of fats, 6.7 g of total dietary fibers (insoluble 1.1 g and soluble 5.6 g), 84.9% of moisture, and 100 kcal of energy (Table 5). The fiber content in reconstitutes soup was if over 50% per serving (200 mL) compared to a daily recommended intake (25 g per day). Consequently, it can be claimed as “high in fiber”. The instant soup product also contributed high β carotene (1489 µg/100 g) and ORAC value (2661 µgTE/100 g). The light yellow color, moderately thick, smooth texture, slightly acidic pH, indeed natural odor, and sweet taste were seen as quality characteristics of reconstituted soup. One serving or about 200 mL of instant soup delivered caloric energy of around 200 kcal (1 kcal/g or mL).

### 3.4. Stability of Dehydrated Soup Powder during Storage

Table 6 represents the physicochemical properties and microbial quality of instant soup powder during storage at 25 ± 2 °C for 6 months. The microbial analysis showed that yeast and mold were present at <25 cfu/g throughout the storage for up to 4 months, and thereafter increased to <50 cfu/g until the end of storage. The number of *E. coli* presented < 1.1 MPN/100 g at the beginning until 4 months, after that an increase in number up to <2.2 MPN/100 g was reported until the end of storage. However, no pathogenic bacteria including *Salmonella*, *S. aureus*, *B. cereus*, and *C. perfringen* were detected during 6 months of storage at room temperature. These microorganism results were within the accepted limit and conformed to Food Notification No. 416 (2020).

The β-carotene content and ORAC value were the highest at day 0 which were about 4919 µg/100 g and 8160 µgTE/100 g and continually reduced to 3960 µg/100 g and 7305 µgTE/100 g for 6 m of storage, respectively. The decrease in β-carotene and antioxidant activity during extended room storage could be related to temperature conditions and the oxidation process. The remaining oxygen in the package, product moisture, room temperature, and storage time may positively affect the acceleration of the oxidation reaction [44]. Furthermore, the polyunsaturated fat content in dehydrated soup products may directly cause lipid oxidation and distribute the free radical and oxidation products [45,46]. These substances might influence the chain oxidation reaction and affect the presence of phytonutrients. During extended storage time, moisture, water activity, and the lightness value of the sample were slightly increased (*p* ≤ 0.05), but no significant (*p* > 0.05) changes in pH, a* and b* values were reported. Increasing moisture content during storage may occur due to the penetration of moisture vapor from the atmosphere through the microscopic pores of packaging material. In raged to the health benefit, it was suggested to keep the instant soup powder product for about 4 months at room temperature (25 ± 2 °C).

The developed elderly soup of both ready-to-eat soup and dehydrated soup powder per serving (about 200 mL) provided calory density of approximately 1 kcal/mL based on recommended macronutrient distributions. Meanwhile, 4.91 kcal/g and 1 kcal/g of caloric values were reported in the elderly soup developed by Irwan [25] and Riyad and Rizk [24], respectively. In addition, the developed soup can be claimed to be a good source of dietary fiber, especially for instant soup powder which contains high fiber and β-carotene content and antioxidant activity. Irwan [25] reported the presence of 3380 µg/g of instant soup powder which was higher than the result reported in the present study, 49.19 µg/g. This could be related to the raw ingredients used in the elderly soup preparation; Irwan [25] used the pumpkin and carrot in a ratio of 2:1, whereas we only used pumpkin as one of the ingredients. Using the thermal process was an advantage in extending the product’s shelf life and ensuring safety for consumption (Table 5 and Table 6). Comparing the product stability, the dehydrated soup powder was more stable than ready-to-eat soup because of the lower water activity and moisture content.

## 4. Conclusions

The commonly available agricultural commodities were utilized to prepare ready-to-eat soup and dehydrated soup products for the elderly. The RTE soup formulation which received higher sensory scores consists of brown rice (15 g), pumpkin (32.5 g), sweetcorn (12.5 g), red tilapia (17.5 g), rice bran oil (1.0 g), and water (21.5 g). The developed elderly soup product content includes 105.5 g/100 g of calories, 58:23:20, C:P:F energy distribution ratio, and 1.58 g/100 g of dietary fibers. In addition, both soup types contain a sufficient amount of β-carotene and antioxidant activity. Importantly, the prepared soup products are microbiologically safe for consumption. Moreover, ready-to-eat soup can be stored in refrigerated condition for 4 weeks, whereas instant soup powder can be stored at room temperature for 4 months without losing nutritional value. Hence, the developed ready-to-eat and instant soup powder are a good alternative for the elderly with required daily energy intake. The developed elderly soup product supplement is suggested for consumption at 1–3 servings per day to satisfy the energy requirements. In addition, the developed soup products were prepared with affordable prices and familiar flavors and tastes. A future study will be conducted on soup fortification with added functional ingredients such as folic acid, vitamin B12, vitamin D, etc., that help to promote health or reduce the risk of disease in seniors.

## Figures and Tables

**Table 1:** Soup formulations by varying carbohydrate ingredients based on a recommended macronutrient energy distribution.

Ingredients	Amount (g/100 g)						
	F1	F2	F3	F4	F5	F6	F7
Brown rice	45.2	0	0	23.3	0	27.0	15.0
Pumpkin	0	78.1	0	41.9	30.0	0	32.5
Sweet corn	0	0	40.0	0	25.0	16.2	12.5
Red tilapia	19.8	11.4	17.5	16.3	17.5	18.9	17.5
Rice bran oil	1.1	0.8	1.25	0.9	1.0	1.1	1.0
Water	33.9	9.7	41.25	17.6	26.5	36.8	21.5
Energy per serving (200 mL)	212	160	218	195	202	215	208
Energy ratio, C:P:F	57:23:21	58:22:20	59:21:20	58:23:20	58:23:20	58:22:20	58:23:20

**Table 2:** Sensory analyses of different soup formulations, F1–F7.

Formulations	Acceptance	Taste	Odor	Texture	Color
F1	3.10 ± 0.31 ^a^	2.80 ± 0.89 ^a^	2.80 ± 0.38 ^a^	3.10 ± 0.31 ^a^	3.05 ± 0.39 ^a^
F2	3.40 ± 0.50 ^b^	3.33 ± 0.47 ^b^	3.60 ± 0.68 ^b^	3.10 ± 0.31 ^a^	3.85 ± 0.76 ^b^
F3	3.10 ± 0.31 ^a^	2.85 ± 0.49 ^a^	3.35 ± 0.59 ^b^	3.00 ± 0.00 ^a^	3.50 ± 0.50 ^b^
F4	4.20 ± 0.62 ^c^	4.10 ± 0.79 ^c^	4.10 ± 0.72 ^c^	4.00 ± 0.46 ^c^	4.45 ± 0.51 ^c^
F5	3.90 ± 0.91 ^b^	3.93 ± 1.03 ^b^	3.80 ± 0.62 ^b^	4.05 ± 0.69 ^c^	4.40 ± 0.50 ^c^
F6	3.85 ± 0.59 ^b^	3.83 ± 1.02 ^b^	4.15 ± 0.67 ^c^	3.85 ± 0.93 ^b^	4.25 ± 0.64 ^c^
F7	4.50 ± 0.31 ^c^	4.60 ± 0.41 ^c^	4.50 ± 0.57 ^c^	4.65 ± 0.49 ^c^	4.30 ± 0.57 ^c^

N = 30, Mean ± SD within the same column followed by different lowercase superscripts is significantly different (*p* < 0.05). Five-point hedonic scale: 1—dislike very much, 2—dislike moderately, 3—neither like nor dislike, 4—like moderately, and 5—like very much.

**Table 3:** Proximate, physicochemical, and microbiology quality of selected elderly soup products (formula 7).

Proximate Composition (g/100 g)		Physicochemical Quality		Microbiological Quality	
Carbohydrate	13.80 ± 0.13	β-carotene	1560.83 ± 62.53 µg/100 g	Yeast and mold	<10 cfu/mL
Protein	4.91 ± 0.08	ORAG	3074.31 ± 110.15 µgTE/100 g	Coliform	<1.1 MPN/100 mL
Fat	1.81 ± 0.09	pH	6.52 ± 0.01	*Escherichia Coli*	not detected cfu/mL
Total fiber	1.58 ± 0.20	Viscosity	1.84 × 10^4^ cp	*Salmonella*	not detected cfu/25 mL
Insoluble fiber	1.18 ± 0.09				
Soluble fiber	0.40 ± 0.13	Color value	L* = 72.12 ± 0.11 a* = 5.52 ± 0.03 b* = 56.71 ± 0.06	*Staphylococcus aureus*	not detected cfu/mL
Moisture	85.56 ± 0.06			*Bacillus cereus*	not detected cfu/mL
Energy	105.5 ± 2.12			*Clostridium- perfringens*	not detected cfu/mL

Values are presented as mean ± SD.

**Table 4:** Physicochemical quality and microbiological analyses of ready-to-eat soup product during storage at 5 °C for 6 weeks.

Parameter	Storage Time (Weeks)						
	0	1	2	3	4	5	6
β-carotene, µg/100 g	1560.8 ± 62 ^c^	1549.9 ± 33 ^c^	1518.0 ± 71 ^bc^	1424.1 ± 42 ^abc^	1501.5 ± 28 ^bc^	1381.8 ± 93 ^ab^	1292.9 ± 50 ^a^
ORAC, µg TE/100 g	3074.3 ± 110 ^d^	3097.5 ± 83 ^d^	2884.5 ± 102 ^cd^	2945.6 ± 115 ^cd^	2727.9 ± 83 ^bc^	2557.7 ± 65 ^ab^	2324.4 ± 123 ^a^
pH	6.52 ± 0.01 ^a^	6.51 ± 0.01 ^a^	6.51 ± 0.0 ^a^	6.52 ± 0.01 ^a^	6.52 ± 0.0 ^a^	6.52 ± 0.01 ^a^	6.52 ± 0.01 ^a^
Viscosity ×10^4^ cp	1.81 ± 0.01 ^bc^	1.84 ± 0.03 ^bc^	1.85 ± 0.03 ^c^	1.78 ± 0.03 ^b^	1.78 ± 0.02 ^b^	1.67 ± 0.04 ^a^	1.64 ± 0.02 ^a^
Color values, L*	72.1 ± 0.1 ^c^	71.3 ± 0.7 ^c^	71.4 ± 0.1 ^c^	71.5 ± 0.5 ^c^	68.6 ± 0.1 ^b^	69.6 ± 0.8 ^b^	67.5 ± 0.4 ^a^
a*	5.5 ± 0.1 ^a^	5.6 ± 0.1 ^a^	5.5 ± 0.1 ^a^	5.7 ± 0.1 ^ab^	5.9 ± 0.3 ^ab^	6.0 ± 0.2 ^b^	6.0 ± 0.2 ^b^
b*	56.7 ± 0.1 ^c^	56.8 ± 0.1 ^c^	56.7 ± 0.1 ^c^	56.3 ± 0.2 ^bc^	56.2 ± 0.5 ^bc^	55.8 ± 0.6 ^ab^	55.4 ± 0.2 ^a^
Yeast and mold, cfu/mL	<10	<10	<25	<25	<25	<50	<50
Coliform, MPN/100 mL	<1.1	<1.1	<1.1	<1.1	<1.1	<1.1	<2.2
*E. Coli*, cfu/mL	nd *	nd	nd	nd	nd	nd	nd
Pathogenic bacteria **	nd	-	nd	-	nd	-	nd

Mean ± SD within the same row followed by different lowercase superscripts is significantly different (*p* < 0.05). * nd = not detected, ** Pathogenic bacteria include *Salmonella* (cfu/25 mL), *S. aureus* (cfu/mL), *Bacillus cereus* (cfu/mL), and *C. perfringens* (cfu/mL) to comply with the Food Notification No. 416, 2020.

**Table 5:** Proximate, physicochemical, and microbiological quality of dehydrated soup powder and reconstituted soup for the elderly.

**Dehydrated Soup Powder**					
**Proximate Composition** **(g/100 g)**		**Physicochemical Quality**		**Microbiological Quality**	
Carbohydrate	41.41 ± 0.58	β-carotene	4918.7 ± 88 µg//100 g	Yeast and mold	<25 cfu/g
Protein	14.72 ± 0.16	ORAG	8159.5 ± 74 µgTE/100 g	*Escherichia coli*	<1.1 MPN/100 g
Fat	5.43 ± 0.13	pH	6.63 ± 0.03	*Salmonella*	not detected, cfu/25 g
Total fiber	30.86 ± 0.52	a_w_	0.47 ± 0.003	*S. aureus*	not detected, cfu/g
Insoluble	5.13 ± 0.13	Color value	L* = 59.20 ± 1.15	*B. cereus*	not detected, cfu/g
fiber			a* = 10.87 ± 0.48	*C.* *perfringens*	not detected, cfu/g
Soluble fiber	25.73 ± 0.40		b* = 63.84 ± 0.74		
Moisture	5.08 ± 0.10				
Calorie	274 Kcal				
**Soup reconstituted by adding 150 g or mL of boiled water in about 55 g of soup powder.**					
**Proximate composition** **(g/100 g)**		**Physicochemical quality**		**Appearances**	
Carbohydrate	15.18 ± 0.23	Β-carotene	1489.3 ± 80 µg/100 g	Bright yellow color, moderately thick, smooth texture, sweet flavor like pumpkin, corn, and jasmine rice combined, less fishy smell, and slightly sweet taste	
Protein	5.56 ± 0.56	ORAG	2660.9 ± 58 µgTE/100 g		
Fat	1.92 ± 0.08	pH Viscosity Color value	6.55 ± 0.1 1.74 × 10^4^ cp L* = 84.88 ± 0.66 a* = 5.37 ± 0.37 b* = 52.41 ± 0.52		
Total fiber	6.74 ± 0.09				
Insoluble	1.10 ± 0.08				
fiber					
Soluble fiber	5.64 ± 0.17				
Moisture	84.81 ± 0.06				
Calorie	101 Kcal				

Values are presented as mean ± SD.

**Table 6:** Physicochemical quality and microbiological analyses of dehydrated soup powder during storage at 25 °C for 6 months.

Parameter	Storage Time (Months)						
	0	1	2	3	4	5	6
β-carotene, µg/100 g	4918.7 ± 88 ^d^	4967.1 ± 98 ^d^	4885.8 ± 26 ^d^	4667.3 ± 79 ^c^	4371.7 ± 83 ^b^	4088.9 ± 65 ^a^	3959.7 ± 76 ^a^
ORAC, µgTE/100 g	8159.5 ± 74 ^c^	8140.3 ± 51 ^c^	8083.9 ± 23 ^c^	7809.6 ± 65 ^b^	7684.7 ± 118 ^b^	7427.0 ± 85 ^a^	7305.2 ± 98 ^a^
pH	6.63 ± 0.03 ^a^	6.63 ± 0.02 ^a^	6.63 ± 0.01 ^a^	6.63 ± 0.0 ^a^	6.63 ± 0.01 ^a^	6.64 ± 0.01 ^a^	6.63 ± 0.01 ^a^
Moisture, %	5.08 ± 0.01 ^a^	5.11 ± 0.01 ^a^	5.11 ± 0.01 ^a^	5.17 ± 0.6 ^ab^	5.19 ± 0.01 ^ab^	5.30 ± 0.06 ^bc^	5.32 ± 0.06 ^c^
Water activity	0.472 ± 0.003 ^ab^	0.471 ± 0.001 ^ab^	0.471 ± 0.001 ^ab^	0.470 ± 0.001 ^a^	0.475 ± 0.001 ^b^	0.480 ± 0.002 ^c^	0.479 ± 0.001 ^c^
Color value L*	59.2 ± 1.2 ^ab^	60.2 ± 0.6 ^b^	58.9 ± 0.3 ^ab^	57.6 ± 0.6 ^ab^	56.6 ± 0.9 ^a^	55.5 ± 1.1 ^a^	55.3 ± 0.9 ^a^
a*	10.9 ± 0.5 ^a^	10.8 ± 0.5 ^a^	10.8 ± 0.3 ^a^	10.8 ± 0.4 ^a^	11.2 ± 0.2 ^a^	11.5 ± 0.4 ^a^	11.5 ± 0.2 ^a^
b*	63.8 ± 0.7 ^a^	63.8 ± 0.4 ^a^	63.5 ± 0.3 ^a^	62.7 ± 0.6 ^a^	61.9 ± 0.7 ^a^	60.6 ± 1.4 ^a^	61.0 ± 1.3 ^a^
Yeast & Mold, cfu/g	<25	<25	<25	<25	<25	<50	<50
E.coli, MPN/100 g	<1.1	<1.1	<1.1	<1.1	<1.1	<2.2	<2.2
Pathogenic bacteria **	nd *	-	nd	-	nd	-	nd

Mean ± SD within the same row followed by different lowercase superscripts is significantly different (*p* < 0.05). * nd = not detected, ** Pathogenic bacteria include *Salmonella* (cfu/25 g), *S. aureus* (cfu/g), *Bacillus cereus* (cfu/g), and *C. perfringens* (cfu/g) to comply with Food Notification No. 416, 2020.

## Data Availability

The datasets generated for this study are available on request to the corresponding author.

## Supplementary Materials

The following supporting information can be downloaded at: https://www.mdpi.com/article/10.3390/foods12081680/s1. 2.4 S microbiological quality; Table S1: Proximate of selected agricultural commodities used to formulate the elderly soup.
