# Supplementary material for: Formulation of Ready-to-Eat Soup for the Elderly: Nutritional Composition and Storage Stability Study
Source: Foods. 2023 Apr 18;12(8):1680. doi: 10.3390/foods12081680 (PMC10138097; doi:10.3390/foods12081680)
Supplement: Supplementary file 1 [file foods-12-01680-s001.zip › foods-2309331-supplementary.pdf]

2.4S. Microbiological quality

For pathogenic microorganisms, *Salmonella* was conducted using the method of ISO 6579 – 1 (2017). Briefly, 10 mL of sample was transferred into 50 mL of 0.1% buffered peptone water and incubated at 36 ± C°, 18 ± 2 hr. 0.1 mL of solution was tipped in 10 mL of Rappaport-Vassiliadis medium with soya (RVS broth), and 1 mL was pipetted into 10 mL Muller-Kauffmann tetrathionate-novobiocin (MKTTn broth). The RVS and MKTTn broth were incubated at 41.5 ± 1°C and 36 ± 1°C for 24 ± 3 hr, respectively. The selection on agar media, 1 loop of a selective liquid media was streaked on Xylose Lysine Deoxycholate agar (XLD agar) and Hektoen enteric (HE) agar, and incubated at 36 ± 1°C for 24 ± 3 hr. The specific colony of *Salmonella* spp. was observed.

Whereas, *Staphylococcus aureus, Bacillus cereus, Clostridium perfringens,* and *Clostridium botulinum* were analyzed following the Bacteriological Analytical Manual (BAM) [13]. To determine the presence of *Staphylococcus aureus*, 0.1 mL of dilution sample was spread on baird parker agar added with 1% egg yolk and potassium tellurite solution, and incubated at 37 ± 1°C for 45-48 hr. The suspect S. aureus colonies was inoculum on trypticase soy agar (TSA), and incubated at 37 ± 1°C, 24 hr., then following confirmation by chemical reaction test (e.g., coagulase test, catalase activity, anaerobic utilization of glucose, oxidase test, voges-proskauer production, methyl red test and anaerobic utilization of mannitol.

Bacillus cereus count was determined by Transferring 1 mL of dilution sample into mannitol-egg yolk-polymyxin (MYP) agar plate, 3 plates at 0.3 mL, 0.3 mL, and 0.4 mL, and incubate plates at 37 ± 1°C, 18-24 hr.

Clostridium perfringens count was determined by 1 mL of dilution sample was pipetted into10 mL of cooked meat medium and incubated under anaerobic at 35 ± 1°C, 24-48 hr. Inoculating 1 loop of this medium on tryptose sulfite cycloserine egg yolk agar (TSC egg yolk) and incubated in anaerobic jar at 35 ± 1°C, 24 hr. Confirmation the colony of C. perfringens by nitrate reduction, motility and spore-forming.

While *Clostridium botulinum* presence was estimated by transferring 1-2 g of food sample into 2 tubes of 15 mL cooked meat medium and 2 tubes of 15 mL tryptone peptone glucose yeast extract (TPGY broth), and incubate at 35°C and 28°C for 5 days, respectively. Note the turbidity, gas production, and digestion of meat particles. If there is not found it should continue to incubate for 10 days. In case, the positive resulted; 1 mL culture medium was mixed with 1 mL absolute ethanol passed through 0.45 μm membrane filter and incubated at room

temperature for 1 hr. Inoculating 1 loop of this culture on anaerobic egg yolk agar and incubated at 35 ± 1°C for 48 hr. in anaerobic jar.

Table S1. Proximate of selected agricultural commodities used to formulate the elderly soup.

Ingredients	Amount (g/100 g, wet basis)					
	**Carbohydrate**	**Protein**	**Fat**	**Dietary fiber**	**Ash**	**Moisture**
Brown rice, cooked	29.94 ± 1.05^{b}	3.22 ± 0.21^{b}	1.30 ± 0.10^{c}	1.87 ± 0.21^{c}	0.49 ± 0.05^{c}	65.05 ± 0.11^{c}
Pumpkin, steamed	11.22 ± 1.56^{c}	1.31 ± 0.08^{bc}	0.32 ± 0.03^{d}	3.0 ± 0.16^{b}	1.02 ± 0.07^{a}	86.13 ± 0.15^{a}
Sweet corn, steamed	34.92 ± 2.43^{a}	3.28 ± 0.15^{b}	1.20 ± 0.05^{c}	5.53 ± 0.31^{a}	0.63 ± 0.03^{b}	59.97 ± 0.12^{d}
Red tilapia, steamed	NC	20.38 ± 1.78^{a}	2.46 ± 0.11^{b}	NA	1.14 ± 0.06^{a}	76.05 ± 0.07^{b}
Rice bran oil	NC	NA	99.74 ± 0.17^{a}	NA	0.15 ± 0.01^{d}	0.10 ± 0.00^{e}

mean ± SD, n=3

NC = Not count and NA = Not analyzed
